# Clinical Notes upon Parturition

**Published:** 1887-10

**Authors:** E. A. Ballard

**Affiliations:** Chicago; Bureau of Obstetrics, etc., I. H. A.


### CLINICAL NOTES UPON PARTURITION.

E. A. Ballard, M. D., Chicago.

(Bureau of Obstetrics, etc., I. H. A.)

Aconite: In labor with her first child. Pains attended with great restlessness; throwing herself from side to side and crying, "Oh! let me die! Let me die!" One dose of Aconite quieted her in five minutes, and child was born a few minutes later.

Calc. carb.: Last part of sixth month of her second pregnancy. Labor pains continued through the day. In the evening they were frequent, hard, downward pressing, mostly in front, with a free, bright show. Os dilated so as to readily admit the finger. During a pain she cried, "Hold me down! Hold me down!" Analyzing this symptom, I found that the stomach was greatly distended, standing out like half a bladder, with a sensation during pains as if that part of her body were rising. One dose controlled the case at once. At the termination of her seventh month she was again threatened with miscarriage. Pains indefinable, not near so hard as before. Anxiety, restlessness, changing from bed to chair or wobbling about, dislike to be alone, cold perspiration. One dose of Arsenic, and an easy labor at full term.

Nux-vomica: Besides the well-known indication, desire for stool or urination with every pain, which I have repeatedly verified, we may find the pains wholly or principally in the back, and during the pains the patient must stand or walk about, likes to have her back rubbed. One lady has in two different labors quickly felt the benign effects of this remedy after twelve hours of do-nothing pains.

Pulsatilla: Regular, hard, downward-pressing pains at beginning of fifth month of pregnancy. Sepia did no good. Found that every pain was accompanied by loss of breath. Remembering to have read in Lippe's *Repertory*, "Difficult respiration accompanies diseased conditions in parts not involved in the act of breathing, Puls.," I gave one dose, which quickly righted matters.

Another case, patient had between pains shivering and chattering of teeth without chilliness. This started each time from anterior middle portion of right thigh, where there was also a sensation of shivering. No relief following the exhibition of Actea r., I sat down to consult my repertory, when I noticed that there was a disposition to weep with every pain. A few minutes after taking a dose of Puls. all these symptoms ceased, and the pains became expulsive and terminated the labor in about twenty minutes. The patient described the effect of the remedy as that of a large ball which went straight down and pushed the child out without any effort on her part.

Unless another remedy is plainly indicated I always give a dose of Arnica at the end of labor. I have used it in the low, medium, and higher potencies. Since using the higher I have had less trouble with after-pains, but I am not certain that the change was not due to the exhibition of the indicated remedy during labor, for in many such cases I have marked the absence of these pains, while in other cases they were promptly controlled by the remedy that had proven homœopathic to the labor pains. In one case where the after-pains were excited by child-nursing, slamming a door, or any sudden noise, Arsenic cured.

